# Size-Controllable Prussian Blue Nanoparticles Using Pluronic Series for Improved Antioxidant Activity and Anti-Inflammatory Efficacy

**DOI:** 10.3390/antiox11122392

**Published:** 2022-12-02

**Authors:** Hyeryeon Oh, Jin Sil Lee, Daekyung Sung, Siyoung Yang, Won Il Choi

**Affiliations:** 1 Center for Bio-Healthcare Materials, Bio-Convergence Materials R&D Division, Korea Institute of Ceramic Engineering and Technology, Cheongju 28160, Republic of Korea; 2 School of Materials Science and Engineering, Gwangju Institute of Science and Technology, Gwangju 61005, Republic of Korea; 3 Department of Pharmacology, Ajou University School of Medicine, Suwon 16499, Republic of Korea

**Keywords:** Prussian blue, Pluronic, reactive oxygen species, antioxidant, anti-inflammation, wound healing

## Abstract

Prussian blue (PB) is a metal cluster nanoparticle (NP) of cyanide-bridged iron(II)–iron(III) and exhibits a characteristic blue color. Its peroxidase-, catalase-, and superoxide-dismutase-like activities effectively remove excess reactive oxygen species that induce inflammation and tumorigenesis. However, the dispersion of PB NPs is not sufficiently stable for their application in the biomedical field. In this study, we developed Pluronic-stabilized Prussian blue nanoparticles (PB/Plu NPs) using a series of Pluronic triblock copolymers as a template material for PB NPs. Considering the hydrophilic–lipophilic balance (HLB) values of the Pluronic series, including F68, F127, L35, P123, and L81, the diameters of the PB/Plu NPs decreased from 294 to 112 nm with decreasing HLB values. The smallest PB NP stabilized with Pluronic P123 (PB/PP123 NP) showed the strongest antioxidant and anti-inflammatory activities and wound-healing efficacy because of its large surface area. These results indicated that the spatial distribution of PB NPs in the micelles of Pluronic greatly improved the stability and reactive oxygen species scavenging activity of these NPs. Therefore, PB/Plu NPs using U.S.-FDA-approved Pluronic polymers show potential as biocompatible materials for various biomedical applications, including the treatment of inflammatory diseases in the clinic.

## 1. Introduction

In living organisms, the production and scavenging of reactive oxygen species (ROS) are strongly controlled by the antioxidant defense system. ROS are highly reactive oxygen derivatives and mainly include superoxide (O_2_^•−^), hydroxyl radicals, hydrogen peroxide (H_2_O_2_), and hypochlorite [1]. These species are generally regulated in an equilibrium state to protect the body from invading pathogens and are involved in physiological processes, such as wound healing and tissue repair [2,3]. However, abnormal overexpression of ROS causes oxidative stress, leading to tissue degeneration and various human diseases [4,5,6]. Researchers have developed several antioxidant nanoparticles (NPs) that control oxidative stress by acting through chemical mechanisms analogous to in vivo redox reactions. For example, cerium NPs mimic antioxidant enzymes such as superoxide dismutase and catalase. Cerium can transfer O_2_^•−^ to H_2_O_2_ by exchanging its transition states [7]. Similarly, selenium and vanadium NPs are potent antioxidant materials because of their glutathione-peroxidase-mimic capacity [8]. Recently, Zhang et al. reported the peroxidase-, catalase-, and superoxide-dismutase-like activities of Prussian blue (PB) NP [9], a mixed-valent iron cyanide complex, Fe_4_^III^[Fe^II^(CN)_6_]_3_, that exists in two transition states and shows strong antioxidant activity [10]. Thus, it exerts therapeutic effects on inflammatory diseases, such as colitis and ischemic stroke, by regulating ROS levels [11,12]. Although further study is required to clarify the exact mechanism of PB’s anti-inflammatory activity, the antioxidant and anti-inflammatory effects of PB have been found to be beneficial for cutaneous wound healing [13]. There have been several reports about the wound-healing efficacy of antioxidant and anti-inflammatory materials. Considering that an excessive ROS level interrupts the healing process, it was reported that redox homeostasis is essential in wound healing [14]. Overproduction of NO results in cytotoxicity and inflammation as an important inflammatory agent. Luo et al. reported that acute inflammation inhibits M1-to-M2 macrophage polarization for tissue regeneration and differentiation [15]. Thus, overexpressed NO inhibitor or scavenger promotes cell migration and proliferation.

PB NPs have been traditionally used as dyes [16] as well as in electrochromic devices [17,18,19] and biosensors [20,21] because of their characteristic blue color and unique electrochemical properties [22]. These NPs have also been approved by the U.S. FDA and have gained attention in the biomedical field as biocompatible antidotes for radioactive thallium and cesium [23]. Although PB NPs are widely used in biomedical fields, their application remains challenging because of their instability in biological buffers and long-term aqueous solutions [24]. Thus, several polymers have been used as stabilizing agents to improve the stability of PB NPs, such as poly(vinylpyrrolidone), chitosan, and polyethylene glycol [25,26,27]. The stabilizing agents are important for preparing highly dispersible PB NPs. Furthermore, they can control the growth of PB NPs with unique size- and shape-dependent properties.

Pluronic may be an excellent candidate as a stabilizing agent for PB NPs. This biocompatible copolymer is composed of one poly(propylene oxide) or two poly(ethylene oxide) blocks [28]. More than 50 types of Pluronic are commercially available, and their properties can be determined based on their block lengths and molecular weights [29]. Pluronic series are characterized by their hydrophilic–lipophilic balance (HLB) value, which is a fractional ratio of the lipophilic region to the hydrophilic region of an amphiphilic molecule. This value is critical to stabilize and optimize the characteristics of metal nanoparticles [30,31]. However, to the best of our knowledge, Pluronic series have not been used as stabilizing materials to improve and optimize the stability of PB NPs.

In this study, we developed Pluronic-stabilized PB NPs (PB/Plu NPs) with easy size control, as well as improved stability and efficacy, by using different types of Pluronic (F68, F127, L35, P123, and L81) as templates, and the resulting PB/Plu NPs were denoted by the type of Pluronic series (e.g., PB/PF68 NP for PB NPs developed with Pluronic F68). The physicochemical characteristics of PB/Plu NPs with different HLB values of the Pluronic triblock copolymers were analyzed, and the influence of templating materials on the antioxidant activity of PB/Plu NPs was assessed both in situ and in vitro. The migration and proliferation of fibroblasts via ROS scavenging and anti-inflammation were also evaluated.

## 2. Materials and Methods

### 2.1. Materials

The Pluronic series (F68, F127, L35, P123, and L81), potassium ferricyanide, iron(II) chloride tetrahydrate, 2,2-diphenyl-1-picrylhydrazyl (DPPH), ethylenediaminetetraacetic acid (EDTA), phosphate-buffered solution (1 M, pH 7.4), 2-thiobarbituric acid, trichloroacetic acid, and lipopolysaccharides from Escherichia coli O111:B4 (LPS) were purchased from Sigma-Aldrich (St. Louis, MO, USA). Methanol, H_2_O_2_ (30%), and L-ascorbic acid were obtained from SK Chemical (Seongnam, Republic of Korea), Junsei Chemical Co. (Tokyo, Japan), and TCI (Tokyo, Japan), respectively. Iron(III) chloride (FeCl_3_) and 2-deoxy-D-ribose (99%) were purchased from Alfa Aesar (Ward Hill, MA, USA).

In vitro cell experiments were carried out in Dulbecco’s modified Eagle’s medium (DMEM; Gibco, Grand Island, NY, USA), supplemented with fetal bovine serum (FBS) from Gibco and antibiotic-antimycotic (AA) from Thermo Fisher Scientific (Waltham, MA, USA). The mouse embryonic fibroblast cell line NIH 3T3 and the murine macrophage cell line RAW264.7 were obtained from Korean Cell Line Bank (Seoul, Republic of Korea). 2′,7′-Dichlorodihydrofluorescein diacetate (H_2_DCFDA) was purchased from Invitrogen (Carlsbad, CA, USA). Griess reagent for colorimetric detection of nitric oxide (NO) was purchased from Abcam (Cambridge, UK). Deionized (DI) water and phosphate-buffered saline (PBS) were obtained from Hyclone (Logan, UT, USA). All chemicals were used without further purification.

### 2.2. Preparation of PB/Plu NPs

Stabilized PB NPs were synthesized using a biocompatible polymer in a manner similar to that in our previous study [32]. The Pluronic series was dissolved in 3 mL of deionized (DI) water at a concentration of 50 mg/mL and then mixed with 1 mL of potassium ferricyanide solution (5 mM) by stirring at 400 rpm. After 30 min, 1 mL of iron chloride tetrahydrate solution (5 mM) was slowly added dropwise. Following the immediate appearance of a blue color, the reaction mixtures were stirred for 1 h at 550 rpm. To prepare bare PB NPs, solutions of potassium ferricyanide and iron chloride tetrahydrate were added to 3 mL of DI water without Pluronic. The as-synthesized PB NP and PB/Plu NPs were spin-filtrated for purification using an Amicon Ultra-15 centrifugal filter (molecular weight cutoff 100 kDa; Merck Millipore, Billerica, MA, USA) and then freeze-dried for 3 days. Lyophilized nanoparticles were stored at −20 °C until use.

### 2.3. Characterization of PB/Plu NPs

The PB/Plu NPs were analyzed to determine their hydrodynamic diameter, polydispersity index (PDI), and zeta potential using a Zetasizer (ELSZ-2000, Otsuka Electronics Co., Ltd., Osaka, Japan). The typical UV–Vis absorption peak of the PB was analyzed with a UV–Vis spectrophotometer (Mega900, Scinco, Seoul, Republic of Korea), and NP morphology was observed by transmission electron microscopy (JEM-2100Plus HR, JEOL, Tokyo, Japan). Fourier-transform infrared spectrophotometry (FT/IR-460 plus, Jasco, Tokyo, Japan) of the PB NPs was performed before and after Pluronic coating.

### 2.4. Stability of PB/Plu NPs

The PB NP and PB/Plu NPs in aqueous solution (10 mg/mL, DI water) were stored in an incubator at 37 °C and 100 rpm. The NPs were then monitored for 4 weeks to detect any changes in their appearance and physicochemical properties without partial aggregation. The hydrodynamic diameter, PDI, and zeta potential of the NPs were analyzed at 0, 1, 2, and 4 weeks using a Zetasizer. In addition, the stability of the PB/PP123 NP among the NPs in a serum-containing biological buffer was analyzed prior to in vitro assays. The NPs in PBS supplemented with 10% FBS were stored in an incubator at 37 °C and 100 rpm. The hydrodynamic diameter and PDI of the NPs were analyzed after 0, 1, 3, 5, and 7 days of storage.

### 2.5. In Situ Antioxidant Activity of PB/Plu NPs

DPPH and deoxyribose assays were carried out to analyze the radical scavenging activity of PB NPs and PB/Plu NPs [32]. First, the light-sensitive DPPH radical was dissolved in methanol at a concentration of 5 mM in the dark. Next, 150 μL of DPPH solution was reacted with the same volume of each sample solution dissolved in DI water at a concentration of 10 mg/mL. To prepare the control group, the radical solution was added to 150 μL of DI water, which had low antioxidant activity. The reaction mixtures were stored at 25 °C with minimal exposure to light, and their UV–Vis absorbance at a wavelength of 515 nm was measured with a microplate reader (VICTOR X5, PerkinElmer, Waltham, MA, USA). The resulting values were used with Equation (1) to calculate the antioxidant activity of the NPs.

(1) $$Antioxidant\;activity\;\left(\%\right)=\left[\frac{\left(∆A_{515}\;of\;control-∆A_{515}\;of\;sample\right)}{∆A_{515}\;of\;control}\right]\times 100$$

Next, the hydroxyl radical scavenging activity of the NPs was evaluated in a deoxyribose assay. Hydroxyl radicals were generated in a reaction containing the following reagents: EDTA (0.1 mL, 0.1 mM), FeCl_3_ (0.1 mL, 0.1 mM), H_2_O_2_ (0.1 mL, 1 mM), deoxyribose (0.1 mL, 3.75 mM), potassium phosphate buffer (0.5 mL, 20 mM), and ascorbic acid (0.1 mL, 0.1 mM). Bare PB NP and PB/Plu NPs were dissolved in DI water at 1 mg/mL, and then 1 mL of each sample solution was added to the hydroxyl radical solution. The control was prepared in DI water as a substitute for the sample solution, whereas the blank was prepared without either H_2_O_2_ or sample solution. After incubation for 1 h at 37 °C, 1 mL of 2-thiobarbituric acid (1% *w*/*v* in 50 mM NaOH) and 1 mL of trichloroacetic acid (2% *w*/*v* in DI water) were added to terminate the reaction. A pink chromogen appeared after heating the reaction mixtures at 85 °C for 20 min, and then absorbance was measured at a wavelength of 515 nm with a microplate reader. The hydroxyl scavenging activity of PB NP and PB/Plu NPs was calculated using Equation (2).

(2) $$Antioxidant\;activity\;\left(\%\right)=\left[\frac{\left(∆A_{515}\;ofsample-∆A_{515}\;of\;blank\right)}{\left(∆A_{515}\;ofcontrol-∆A_{515}\;of\;blank\right)}\right]\times 100$$

### 2.6. In Vitro Cytotoxicity

The cytotoxicity of PB/PP123 NP among the PB/Plu NPs was evaluated using NIH 3T3 mouse embryonic fibroblasts cultured in DMEM supplemented with 10% FBS and 1% AA. Approximately 10,000 cells were seeded into each well of a 96-well plate and incubated overnight in a humidified atmosphere of 5% CO_2_ at 37 °C. PB/PP123 NPs at concentrations of 0–5 mg/mL were treated and incubated for 1 day. Cell viability was analyzed after adding Cell Counting Kit-8 solution (Dojindo Laboratories, Kumamoto, Japan) diluted to 10% in DMEM. After 1 h of incubation, the absorbance of the formazan produced by viable cells was measured with a microplate reader at a wavelength of 450 nm, and cell viability was calculated using Equation (3).

(3) $$Cell\;viability\;\left(\%\right)=\left(\frac{∆A_{450}\;of\;sample}{∆A_{450}\;of\;control}\right)\times 100$$

### 2.7. In Vitro Antioxidant Activity

NIH 3T3 cells cultured in DMEM containing 10% FBS without AA were stimulated with the oxidative stress agent H_2_O_2_ to generate ROS. The decrease in ROS levels after treatment with PB/PP123 NP was analyzed to determine the in vitro antioxidant activity of PB NPs. Briefly, the cells were seeded into a 96-well plate at a density of 10,000 cells per well and incubated overnight. Next, 100 µL of each PB/PP123 NP at concentrations of 0–1000 ng/mL and H_2_O_2_ (5 μM) were added to the cells and incubated for 8 h. H_2_O_2_ was not added to the control. After removing the sample solutions by washing with PBS, the cells were treated with the fluorescent ROS indicator H_2_DCFDA (10 μM) for 90 min with minimal exposure to light. Fluorescence from dichlorofluorescein oxidized by in vitro ROS was measured at excitation and emission wavelengths of 485 and 535 nm, respectively, using a microplate reader.

### 2.8. In Vitro Anti-Inflammatory Activity

The inhibition of NO production from LPS-stimulated RAW264.7 cells was evaluated after treatment with PB/PP123 NP. Briefly, RAW264.7 cells were cultured in DMEM containing 10% FBS without AA. They were seeded into a 96-well plate at a density of 10,000 cells per well. After incubation, various concentrations of PB/PP123 (0, 0.01, 0.1, and 1 mg/mL) and LPS (100 ng/mL) were applied for 1 day in a humidified incubator with 5% CO_2_. Next, 100 μL of each of the supernatants was collected and reacted with an equal volume of Griess reagent. A microplate reader was used to measure the absorbance of the mixtures at a wavelength of 540 nm to determine the NO concentration. The test group without LPS treatment was used as a control with the lowest absorbance.

### 2.9. In Vitro Wound-Healing Activity

A scratch wound-healing assay, which is a simple and economical method, was conducted to evaluate cell migration and proliferation [33]. NIH 3T3 fibroblasts were seeded into a 24-well plate (200,000 cells/well). When the cells reached confluency and had formed a monolayer, a scratch was made using a sterile P1000 micropipette tip. PB/PP123 NPs at different concentrations (0, 0.01, 0.1, 0.5, and 1 mg/mL) were added to the cells after removing cell debris by washing with DMEM. During incubation at 37 °C for 24 h, microscopic images were acquired at 0, 4, 8, and 24 h using a DMi1 light microscope (Leica, Wetzlar, Germany). ImageJ software 1.8.0 (NIH, Bethesda, MD, USA) was used to calculate the wound gap distance.

### 2.10. Statistical Analysis

All experiments were performed in triplicate (*n* = 3). The resulting data are expressed as the mean ± standard deviation. Student’s *t*-test and one-way *ANOVA* were used to compare the in vitro results. A *p*-value < 0.05 was considered statistically significant. Therefore, the significance symbols (namely, #, *, **, and ***) are used to indicate *p* > 0.05, *p* < 0.05, *p* < 0.01, and *p* < 0.001, respectively.

## 3. Results and Discussion

### 3.1. Preparation and Characterization of PB/Plu NPs

**Figure 1:**
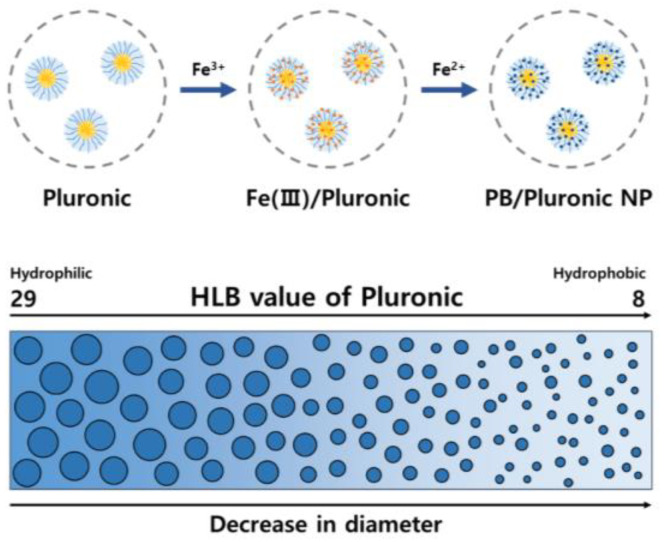
Schematic preparation of novel Prussian blue nanoparticles stabilized by Pluronic polymers (PB/Plu NPs). HLB, hydrophilic–lipophilic balance.

Pluronic series including F68 (HLB 29), F127 (HLB 22), L35 (HLB 19), P123 (HLB 8), and L81 (HLB 2) were used as polymer templates to prepare stabilized PB NPs (Figure 1). These Pluronic types are composed of a hydrophobic propylene oxide and two hydrophilic ethylene oxide units that are electron-rich and form complexes with Fe^3+^ [34]. Fe^3+^/Pluronic complexes function as active sites when iron chloride tetrahydrate solution is added. Thus, the Pluronic-templated PB NPs (PB/Plu NPs) were prepared with PB NPs intercalated in Pluronic micelles. The micellar behavior of Pluronic depends on the ratio of propylene oxide to ethylene oxide units, known as the HLB [35].

**Figure 2:**
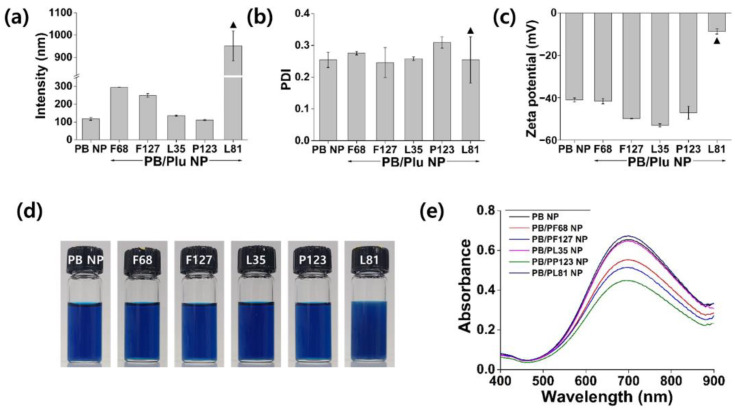
Characterization of Prussian blue nanoparticles (PB NPs) and Pluronic-stabilized Prussian blue nanoparticles (PB/Plu NPs). (**a**) Hydrodynamic diameters; (**b**) polydispersity index (PDI); (**c**) zeta potentials; (**d**) photographs of the PB NP and templates used (Pluronic series including F68, F127, L35, P123, and L81); (**e**) UV–Vis absorption spectra of PB NP and PB/Plu NPs. ▲ indicates partial aggregation of nanoparticles.

With decreasing HLB values of Pluronic, the hydrodynamic diameter of PB/Plu NPs tended to decrease. Although the size of bare PB NP was approximately 118 nm, the diameters of PB NPs stabilized by Pluronic decreased from 294 to 111 nm with decreasing HLB values (Figure 2a). This phenomenon resulted from the more hydrophilic nature of Pluronic with higher HLB values [36]. However, Pluronic L81 (PL81), which has the lowest HLB value, did not stabilize PB NPs in aqueous solution because of its high lipophilicity, leading to a large size with partial aggregation. The PDI of all NPs except PB/PL81 NP was below 0.3, indicating a uniform distribution of the particle size (Figure 2b). In the surface charge analysis, bare PB NPs exhibited a negative surface charge of approximately −41 mV, whereas PB/Plu NPs shifted to a more negative value because of the spatial distribution of PB NPs in the Pluronic matrices (Figure 2c). The shift was greater as the hydrodynamic diameter decreased, possibly because of the increased surface area of the smaller NPs. Therefore, the surface charge of partial-aggregated PB/PL81 NPs was near-neutral. Additionally, PB NPs and all PB/Plu NPs showed a blue color because of the charge transfer transition in the Fe^2+^-CN-Fe^3+^ bond (Figure 2d) [37]. However, the blue color of PB/PL81 NPs was blurry in the presence of partial aggregates. The characteristic absorbance peak of PB NPs in the near-infrared region was observed in the UV–Vis spectra of all NPs (Figure 2e). The wavelength of the maximum absorbance was approximately 700 nm, and the corresponding absorbance intensities differed depending on the degree of complexation of PB with Pluronic [38]. The PB/PL35 NPs and PB/PL81 NPs exhibited similar absorbance intensities to those of bare PB NPs. In contrast, the other PB/Plu NPs showed lower absorbance intensities, indicating better interactions of Pluronic with PB NP for stabilization. In particular, PB/PP123 NPs showed the lowest absorbance intensity.

**Figure 3:**
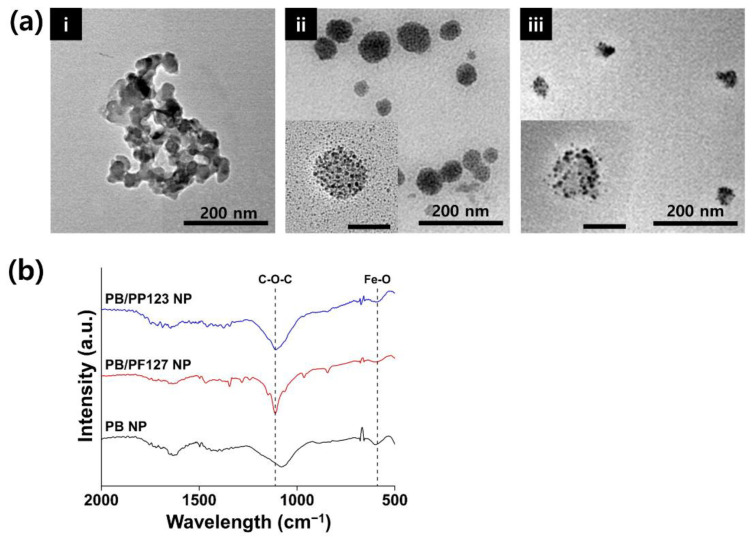
Physicochemical properties of PB NP and PB/Plu NPs. (**a**) Transmission electron microscopy images of (i) PB NP, (ii) PB/PF127 NP, and (iii) PB/PP123 NP (scale bar = 200 nm). Insets are highly magnified images with scale bars of 50 nm. (**b**) Fourier-transform infrared (FT-IR) spectra of PB NP, PB/PF127 NP, and PB/PP123 NP.

According to the transmission electron microscopy images, bare PB NPs aggregated as a cluster with a size of approximately 200 nm because of their instability (Figure 3a). However, PB/PF127 NP and PB/PP123 NP were spherical, with PB NPs intercalated inside the micelles. As characterized by dynamic light scattering, PB/PP123 NP was approximately two-fold smaller than PB/PF127 NP based on the transmission electron microscopy images. In addition, the complexation of PB NPs with Pluronic template was confirmed by Fourier-transform infrared spectroscopy (Figure 3b). Unlike the spectrum of PB NPs, the bands at 588 and 1108 cm^−1^, corresponding to Fe-O stretching and C-O-C vibrations, respectively, appeared after coating with PF127 and PP123 [39]. After coating, the peak at 601 cm^−1^, corresponding to Fe^3+^-CN-Fe^2+^ in the spectrum of bare PB NPs, shifted to 588 cm^−1^ by the Fe-O peak in the PB/Plu NPs [40]. The Fe–O bond suggests an association of the hydroxyl groups of Pluronic with Fe^3+^ in PB. The C-O-C bond was also typically observed from Pluronic, indicating the presence of Pluronic on the surface of PB NPs.

### 3.2. Stability of PB/Plu NPs

**Figure 4:**
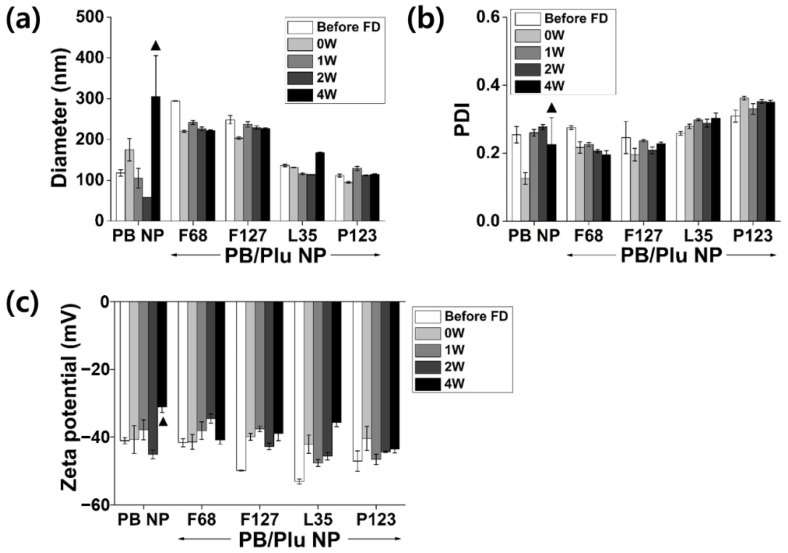
Stability analysis of PB NP and PB/Plu NPs in aqueous solution at 37 °C. (**a**) Hydrodynamic diameters; (**b**) polydispersity index (PDI); (**c**) zeta potential after freeze drying (FD) and 4 weeks of storage at 37 °C. ▲ denotes partial aggregation of the nanoparticles.

PB NP and PB/Plu NPs were successfully lyophilized and easily redispersed in aqueous solution without altering their characteristics (size, PDI, and surface charge) for ease of storage and usage (Figure 4). The hydrodynamic diameter of bare PB NPs noticeably increased with the appearance of partial aggregation after four weeks of incubation, whereas all PB/Plu NPs maintained their initial size. No change in the PDI or zeta potential of PB/Plu NPs was observed during storage. This indicates that the stability of PB NPs was improved after templating with Pluronic polymers. Notably, PB/PP123 NP was also stable in biological buffer without any change in the physicochemical properties of the NPs (Supplementary Figure S1). After a week of storage at 37 °C, both the hydrodynamic diameter and PDI of the PB/PP123 NPs were maintained, whereas bare PB NPs were immediately aggregated in biological buffer. As previously reported, the stability of the metal NPs was effectively enhanced by adding more lipophilic Pluronic [41]. The critical micelle concentration that determines the stability of aqueous Pluronic micelle decreases with increasing fractions of hydrophobic propylene oxide blocks; therefore, PB/PP123 NPs with low HLB values may be more suitable for biomedical applications in which Pluronic micelles are diluted in the body fluid.

### 3.3. In Situ Antioxidant Activity of PB/Plu NPs

**Figure 5:**
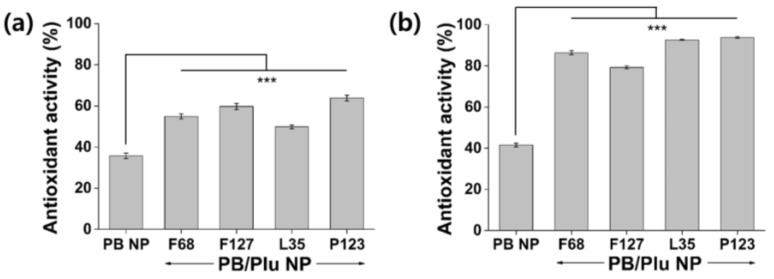
In situ antioxidant activity of PB NP and PB/Plu NPs. (**a**) 2,2-Diphenyl-1-picrylhydrazyl (DPPH) radical scavenging assay and (**b**) hydroxyl radical scavenging assay of PB NP and PB/Plu NPs. *** *p* < 0.001.

The antioxidant activity of PB/Plu NPs was compared with that of bare PB NPs (Figure 5). Templating with Pluronic improved the radical scavenging activity of PB NPs. PB/Plu NPs showed almost two-fold higher radical scavenging activity than PB NPs because the surface area of PB NPs increased via intercalation in the Pluronic micelle (Figure 5a). Absorption of DPPH radicals is more favorable when the surface area is larger [42]. PB/PP123 NPs, with the smallest diameter among the PB/Plu NPs, exhibited the highest antioxidant activity of 64%, as expected. This is consistent with a report by Wang et al., who found that smaller NPs provided a larger surface area for radical scavenging [43]. Similarly, the hydroxyl radical scavenging activity of PB NPs greatly increased after coating with Pluronic (Figure 5b). In particular, the smallest PB/PP123 NP had a remarkably strong antioxidant activity of 94% at a low concentration of 1 mg/mL. Hydroxyl radical scavenging activity was higher than DPPH radical scavenging activity, as PB NP is an effective scavenger of hydroxyl radicals because of its binding affinity for these radicals [10,44].

### 3.4. In Vitro Cytotoxicity and Antioxidant and Anti-Inflammatory Activities

**Figure 6:**
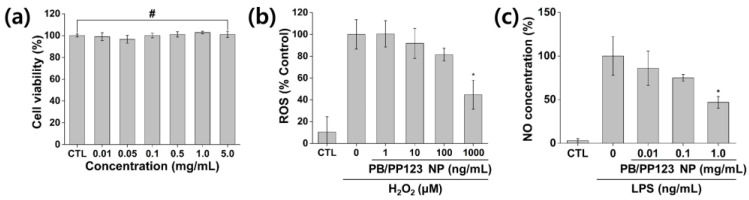
In vitro cytotoxicity, antioxidant activity, and anti-inflammatory activity of PB/PP123 NP. (**a**) Cytotoxicity analysis of 0.01–5 mg/mL NPs, (**b**) antioxidant effect of NPs (control (CTL) indicates the lowest reactive oxygen species (ROS) level), and (**c**) anti-inflammatory efficacy (CTL indicates the lowest nitric oxide level). # *p* > 0.05 and * *p* < 0.05.

Among the PB/Plu NPs, the PB/PP123 NPs, which showed better stability and strong antioxidant activity, were examined to determine their efficacy in an in vitro model. As shown in Figure 6a, the NPs exhibited no cytotoxicity toward mouse embryonic fibroblasts. Accordingly, cell viability was greater than 95% after treatment, even at a high concentration of 5 mg/mL, indicating that PB/Plu NPs can be applied in the biomedical field without causing harmful effects, as both PB NPs and Pluronic are well-known biocompatible materials. In vitro ROS levels were then measured using H_2_DCFDA reagent to determine the in vitro antioxidant activity of PB/PP123 NPs (Figure 6b). The 100% ROS levels in H_2_O_2_-treated NIH 3T3 cells decreased after treatment with the NPs. As expected, increasing amounts of NPs led to stronger antioxidant activity, with effective scavenging of 55% of the ROS (*p* < 0.05). This suggests that PB/PP123 NPs with biocompatible properties can be used as antioxidants in various biomedical fields in vivo.

The efficacy of PB/PP123 NPs on NO inhibition in LPS-stimulated RAW264.7 cells is shown in Figure 6c. When NO production in RAW264.7 cells was induced by LPS (100 ng/mL), the NPs significantly reduced the cellular NO level in a dose-dependent manner (*p* < 0.05). The effective inhibition of NO, a major proinflammatory mediator, indicates that PB/PP123 NP can be used as a prominent anti-inflammatory agent [45]. These results are consistent with those for other anti-inflammatory compounds. Shalini et al. demonstrated that tricin effectively reduced the NO production in a dose-dependent manner by suppressing iNOS expression [46]. Han et al. suggested that histidine is involved in the deactivation of the MAPK and JAK1/STAT3 signaling pathways to effectively reduce NO production in LPS-stimulated RAW264.7 cells [47]. In addition to these anti-inflammatory agents, PB/PP123 NPs are another therapeutic option against inflammatory diseases.

### 3.5. In Vitro Wound-Healing Efficacy

**Figure 7:**
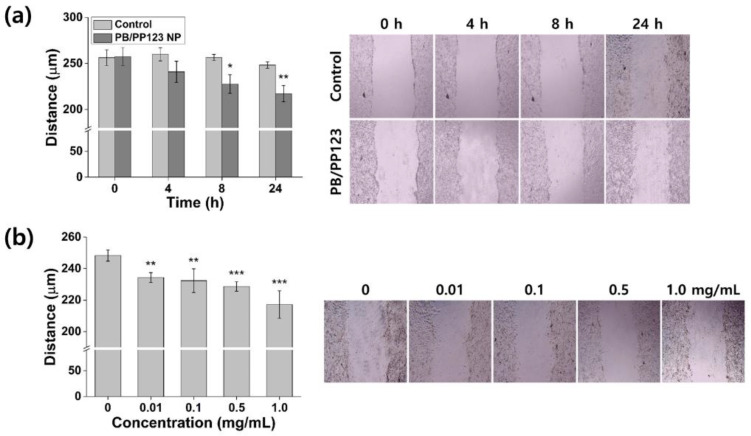
In vitro wound-healing efficacy of PB/PP123 NP. (**a**) Wound closure of NIH 3T3 fibroblast cells after treatment with NPs at different time points, and (**b**) wound-healing activity of NPs at different concentrations after 24 h of treatment (* *p* < 0.05, ** *p* < 0.01, and *** *p* < 0.001).

Damaged tissues produce high levels of ROS that delay wound healing. Therefore, ROS detoxification using ROS-scavenging enzymes can accelerate the effect of wound healing in cells [48]. Similarly, metal oxide NPs such as Al_2_O_3_, CeO_3_, and Y_2_O_3_, which are well-known antioxidant enzyme mimics, were reported to effectively induce wound healing [49]. To determine the efficacy of PB/PP123 NP in wound healing, a monolayer of fibroblast cells that had been damaged by scratching was treated for 24 h (Figure 7). In the control group, only minimal changes to the wound closure were observed; in contrast, the wound size of NP-treated cells decreased from 257 to 217 μm (Figure 7a). The wound distance after 8 or 24 h of treatment was significantly smaller than the initial wound gap (*p* < 0.01 or *p* < 0.001). The wound-healing efficacy increased with increasing concentrations of NPs (Figure 7b; *p* < 0.01) because of the stronger antioxidant activity of the NPs at higher concentrations. Notably, PB/PP123 NP promoted much faster cell migration and proliferation without starvation (Supplementary Figure S2). Therefore, PB/PP123 NPs can result in successful wound healing via their effective ROS-scavenging ability, suggesting the therapeutic potential of this material for various biomedical applications.

## 4. Conclusions

PB NPs were successfully stabilized by spatial distribution in the micelles of Pluronic polymers. Five types of Pluronic with various molecular weights and HLB values conferred PB/Plu NPs with different characteristics. In particular, the diameters of the PB/Plu NPs decreased following stabilization with a more lipophilic Pluronic. PB/PP123 NPs, being the smallest among the PB/Plu NPs, had a large surface area, facilitating the scavenging of ROS radicals. The NPs showed not only remarkable biocompatibility with no cytotoxicity even at a high concentration of 5 mg/mL, but also outstanding antioxidant and anti-inflammatory effects in vitro. Furthermore, the NPs showed favorable wound-healing efficacy because of their strong antioxidant and anti-inflammatory activities, which increased cell migration and proliferation. Therefore, PB/Plu NPs may be useful as antioxidants to protect against ROS-related diseases, including inflammation, in the biomedical field.

## Data Availability

All data analyzed in this study are included in this article and its Supplementary Material.

## Supplementary Materials

The following supporting information can be downloaded at: https://www.mdpi.com/article/10.3390/antiox11122392/s1, Figure S1: Stability analysis of PB/PP123 NPs in a biological buffer. (a) Hydrodynamic diameter and (b) polydispersity index (PDI) of the NPs after a week of storage at 37 °C; Figure S2: In vitro wound-healing efficacy of PB/PP123 NP in FBS-containing cell media without starvation. Wound closure of NIH 3T3 fibroblast cells after 48 h of treatment with NPs at different time points.
