# Supplementary material for: Size-Controllable Prussian Blue Nanoparticles Using Pluronic Series for Improved Antioxidant Activity and Anti-Inflammatory Efficacy
Source: Antioxidants (Basel). 2022 Dec 2;11(12):2392. doi: 10.3390/antiox11122392 (PMC9774457; doi:10.3390/antiox11122392)
Supplement: Supplementary file 1 [file antioxidants-11-02392-s001.zip › antioxidants-2020527-supplementary.pdf]

Supporting Information

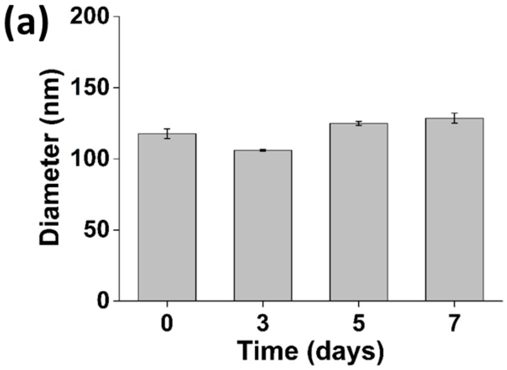

Figure S1. Stability analysis of PB/PP123 NP in a biological buffer. (a) Hydrodynamic diameter and (b) polydispersity index (PDI) of the NP after a week of storage at 37 °C.

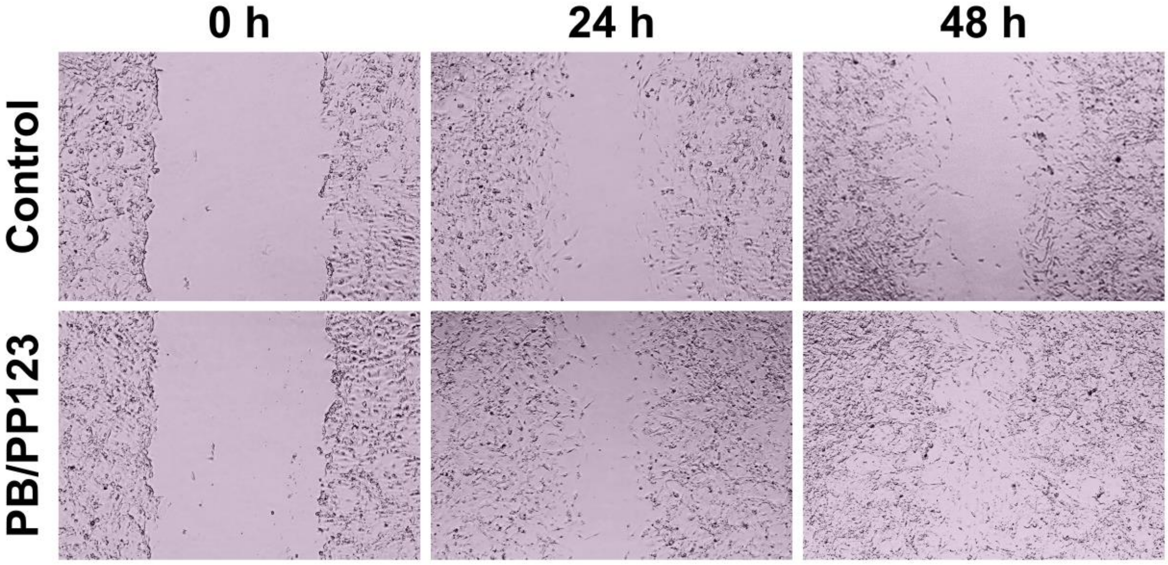

Figure S2. In vitro wound-healing efficacy of PB/PP123 NP in FBS-containing cell media without starvation. Wound closure of NIH 3T3 fibroblast cells after 48 h of treatment with NPs at different time points.
